# Multiple mini-interviews is a predictor of students’ academic achievements in early undergraduate medical years: a retrospective study

**DOI:** 10.1186/s12909-023-04183-7

**Published:** 2023-03-27

**Authors:** Faiza Alam, Ya Chee Lim, Li Ling Chaw, Fazean Idris, Kenneth Yuh Yen Kok

**Affiliations:** PAPRSB Institute of Health Sciences, Universiti Brunei Darussalam, Jalan Tungku Link, Gadong, Bandar Seri Begawan, BE1410 Negara Brunei Darussalam

**Keywords:** Multiple mini-interview, Academic performances, Medical students, Onsite, Online

## Abstract

**Background:**

Our study determined Multiple Mini-Interview (MMI) effectiveness in evaluating specific skill sets based on medical students’ performances during the undergraduate years and compared the academic performances of medical students who appeared for onsite/online MMI.

**Methods:**

A retrospective study of 140 undergraduate medical students between 2016 and 2020 included data on age, gender, pre-university results, MMI scores, and examination results. Appropriate non-parametric tests were applied to compare the students’ MMI and academic performances.

**Results:**

Ninety-eight students from cohorts 12 to 15 had an overall MMI score of 69.0(IQR: 65.0—73.2)/100 and an overall Cumulative Grade Point Average(GPA) of 3.64 (3.42—3.78)/5.0. Spearman’s correlation revealed a significantly positive relationship between MMI and cGPA (rho = 0.23) and GPA from the first 2 semesters (GPA1 rho = 0.25, GPA2 rho = 0.27). This observation was similar to that for station A in the first year (cGPA rho = 0.28, GPA1 rho = 0.34, GPA2 rho = 0.24), and in station B (GPA4 rho = 0.25) and D (GPA3 rho = 0.28, GPA4 rho = 0.24) in the second year. Of twenty-nine cohort16 students, 17(58.6%) underwent online and 12(41.4%) offline modes of MMI assessment, respectively. The overall median MMI score was 66.6(IQR: 58.6—71.6)/100, and the overall median cGPA was 3.45 (3.23—3.58)/5.0. When comparing the median marks of cohort16 groups, the online group scored significantly higher marks for station D than the offline group (*p* = 0.040).

**Conclusion:**

Correspondence between MMI scores and cGPA predicted MMI scoring during student selection and entry process might ensure the success of their academic performance in medical school.

**Supplementary Information:**

The online version contains supplementary material available at 10.1186/s12909-023-04183-7.

## Introduction

Medical practitioners require more than academic abilities to be competent in their line of work; hence assessments of non-cognitive qualities such as empathy and interpersonal skills have been viewed as equally important [1]. Evidence of Multiple Mini Interviews (MMI), a series of stationed whereby candidates being interviewed on a different subject, has been stated as “a non-biased, practical, feasible, reliable, and content-valid admission tool” [2], and the utilization of Multiple Mini Interview (MMI) as a means to assess non-cognitive characteristics have been supported by several validity tests [3]. One such way of assessing the non-cognitive characteristics of the candidates is the Multiple Mini Interview (MMI) [4], where candidates are interviewed in a series of stations, each with a different interviewer.

Eligibility tests and Grade Performance Average (GPA) have been reported to be good pre-admission indicators and authentic medical student selection formats [5]. In the past decade, the demand for assessing complex skills and performances at the time of medical school admission has caused the emergence of MMI as a preferred mode of access in most medical schools globally. The abundance of literature vouches for MMI as a precise method of admittance to evaluate the non-cognitive traits of candidates in medical science fields when compared to traditional interviewing. Furthermore, MMI has been related to good scores obtained from the respective clerkship directors and to the theoretical exams along with the objective structured clinical examinations (OSCE) across different study groups of additional years [6]. Numerous studies advocated the consistency and reliability of the MMI [7] however, there is a dearth of evidence to support the predictive power of MMIs with regard to academic success in the initial years of medical school [6, 8, 9]. Appraising critical thinking, presentation skill, professionalism, and communication skill during MMI may be essential aspects of predicting academic achievements, especially in performance-based subjects.

MMI provides a valid indication of a candidate's non-cognitive characteristics than traditional admissions tools [9]. A strong relationship between practical interpersonal communication skills and healthcare outcomes has been well established. A moderate positive correlation between the average MMI interpersonal score and the communication score on the OSCE was observed [10]. Meanwhile, MMI scores correlated positively with performance-based exams during the first and second years [4, 6]. In particular, critical thinking, presentation, professionalism, and communication skills might be essential competencies in performance-based academic achievement, as well as significant predictors for evaluating post-graduation performances [4].

Critical thinking skills predict academic success during the medical education field's preclinical years [11]. In assessing individual station and student outcomes, Lee et al. (2016) demonstrated the following findings from their MMI results; first, critical thinking and presentation skills assessment stations showed that the score could predict the results of written and performance-based exam in the second-year, but not results of written test in first year [4]. This might be because first-year tests are highly focused on basic medical skills, not medical reasoning. In the second year, knowledge and reasoning-oriented clinical medicine courses were assessed, and findings showed that this particular station’s scores could predict both written and performance-based results.

Second, scores from stations assessing professionalism, including communication skills, self-understanding, and motivation, were most highly correlated with performance-based exams, especially in Doctoring Medical Humanities, which is about developing medical professionalism such as self-awareness, self-confidence, self-regulation, motivation and career choice.

Evidence has clearly shown that MMI is a valuable tool for medical student selection, especially in the areas of critical thinking, professionalism, presentation and communication skills [4]. It predicts medical student performance in clinical clerkships, OSCE and licensing examinations [10, 12, 13]. Hence, our proposed study would evaluate the relationship between individual MMI station scores with results or performances in each module [Health Sciences(HS); Patient care(PC;); Our Community & Professional and personal Development (OCPPD) and Special Study Module (SSM) 1, 2 & 3] to see if specific themes testing a particular skill can be tested to predict student performances in any of the modules.

Amid social distancing, conducting MMI online has become the norm [14]. Online MMI has enabled social distance and eliminated the necessity for travel. Nevertheless, online MMI has been used at the University of Sydney since 2006 for graduate medical and dental programmes [15]. Zoom (Zoom Video Communications, 2011), a digital cloud-based video platform, has been utilised by three reported universities so far [14, 16, 17]. Online MMI has brought about easy access and less expense for applicants but also eliminates unstructured interactions between applicants and interviewers [17]. Online MMI is feasible and acceptable, requiring proficient coordination among team members and a reliable high-speed internet [14]. There have been a number of studies published supporting the feasibility of conducting online MMI for the selection process of undergraduate medical school intake during the pandemic, despite challenges with internet connectivity and familiarity with the use of videoconferencing technology [18, 19]. Advantages of the virtual format included ease of access for faculty and more flexibility, less expense for applicants and save resources [20]. Online MMI and traditional MMI have been reportedly to produce comparable results [21]. This ease of access and low-cost expenditure for applicants of online MMI may be the norm alongside face-to-face when social distancing eased up.

Evidence shows traditional face-to-face MMI usually consists of between 5 and 12 stations in a circuit. As changeover between station to station requires more coordination time, such as leaving a room and then joining another room, four stations were implemented in each circuit [14]. Furthermore, the literature also suggests that online MMI enables an interviewer to have real-time interaction at two physical locations to measure various non-cognitive attributes such as problem evaluation, oral communication or ethical reasoning [22].

**Fig. 1:**
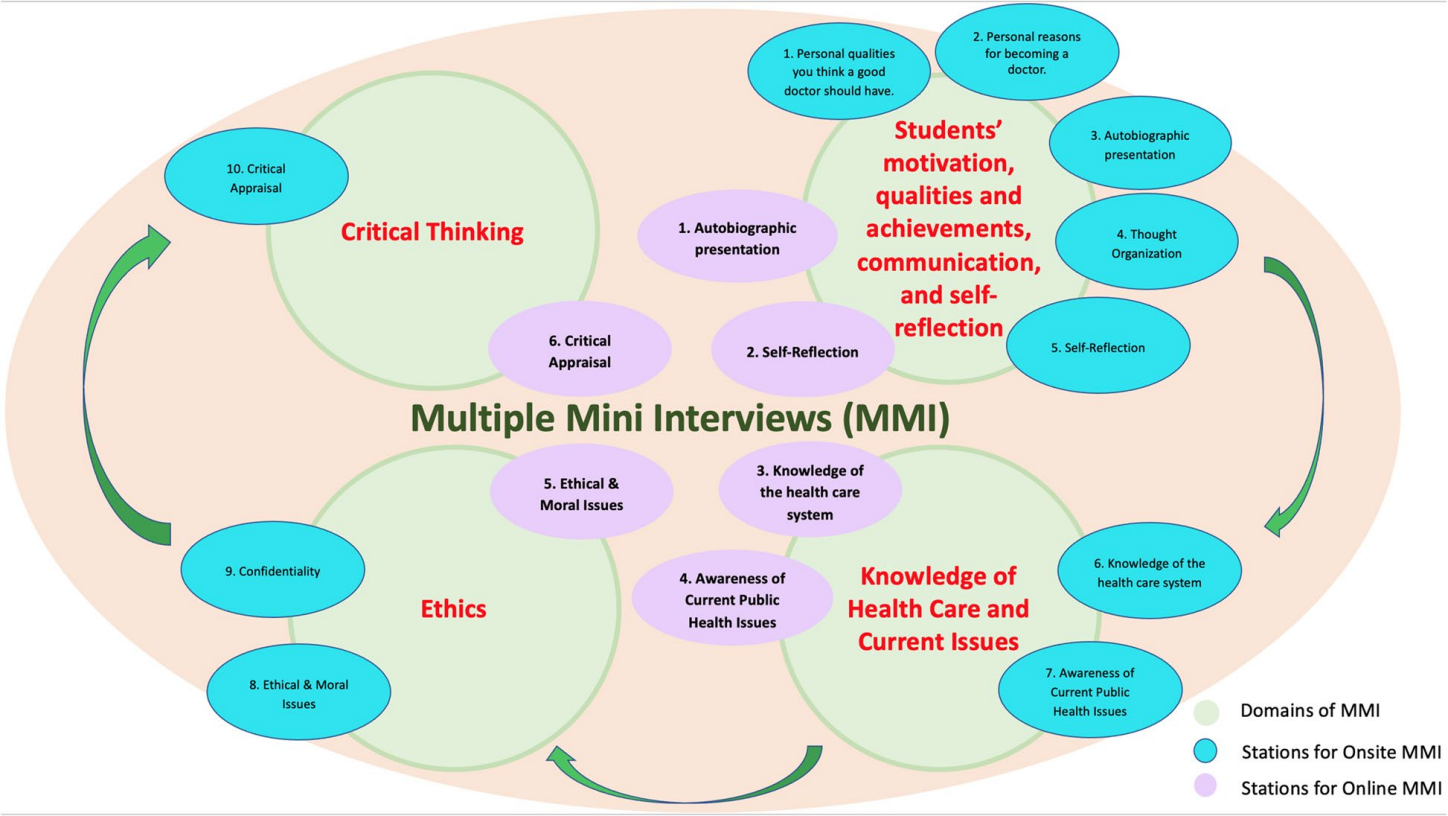
Pictorial presentation of number of Station, Domains and List of Stations Assessed in Onsite and Online MMIs

During the process of onsite and online MMIs held in Universiti Brunei Darussalam (UBD), the same domains were used in the online format, but with lesser stations. Example, the domain on students’ motivation, qualities and achievements, communication, and reflection still maintained the same two stations on “*autobiographic presentation”* and “*reflection”*, while the stations on “*personal qualities you think a good doctor should have”* and “*personal reasons for becoming a doctor”* were assessed in the form of written personal statements. The other domains maintained the same stations and only the confidentiality station was removed from the Ethics domain. Figure 1 summarizes the station domains and list of stations assessed in onsite and online MMIs. As the stations still assessed the overall domains, we feel the results of onsite and online MMI are comparable in terms of the content assessment.

PAPRSB Institute of health Sciences, UBD has offered undergraduate medical education since 2005, where students spend the first three years in the local faculty followed by three years at one of UBD’s partner medical schools in the United Kingdom, Ireland, Australia and Hong Kong. All students successful in the six semesters of the programme will graduate with a Bachelor of Health Science degree and proceed to overseas partner medical schools to pursue the remaining three years of the medical degree. The academic success of the students is measured by the GPA, which is the average of grade points or marks obtained for the modules sat in a particular semester (GPA) but even more so by the cumulative grade point average (cGPA), the average of grade points of all six semesters completed.

The medical programme in PAPRSB Institute of Health Sciences, UBD is a fully integrated course in which four main curricular areas i.e. Health Sciences; Patient Care; Our Community and Personal and Professional Development are studied throughout, with early clinical experience from the start. In addition, students undertake Special Study Modules (SSM 1,2,3) in a project of interest to develop their self-critical approach and gain insight in scientific method, research discipline and presentation of data. The first SSM is a longitudinal attachment, where students are attached over the whole of the first year to a family and conduct regular visits to the family each time with specific goals. The second SSM is a research-based project, while the third SSM involves student groups to work with community members and other organizations in implementing a community-based project.

In the MMI selection criteria, four domains are assessed, which had been designed to relate to students’ expected learning outcomes in the curriculum, ranging from their systematic ability to understand and apply knowledge in the sciences relevant to the application of medicine, to clinical, communication, ethics and research areas. The expected outcomes and its relevance to the curriculum, corresponding to each MMI domain, is summarized in Table 1.

**Table 1:** Station Domains and List of Stations Assessed in Onsite and Online MMIs

Domain	Expected outcomes	Relevance to curriculum
Students’ motivation, qualities and achievements, communication, and self-reflection	Besides intelligence and a strong command of academia as shown by pre-university exam results, qualities such as ambition, dedication, passion, confidence, patience, motivation to learn, discipline, perseverance, good professional communication, teamwork, and altruism are assessed in the MMI. Such aspects are felt to be highly important in the selection process, which may ultimately lead to the success of an individual. While ability to reflect can improve one’s self-regulation and broaden perspective, it can also lead to improved performance, increased motivation and being ready to take on new challenges	• Overall • Health Sciences • Patient Care (Clinical and Communication Skills) • Special Study Module 1 and 3 (Family Case Project, Community Project)
Knowledge of Health Care and Current Issues	Students interested in pursuing medicine would be well-expected to have some knowledge in health care issues and the current health care system. Competent students in this area would predict them to have a better appreciation of community and population medicine including healthcare in the wider community, as well as psychosocial aspects of health and illness. Here, the student’s past shadowing or work experiences in health settings would also be enquired, while their awareness on current issues such as natural disasters and other world events not only indicates their maturity and appreciation of global issues but can serve to potentially lead them to think strategically and proactively to be an empowered, holistic clinician	• Patient Care (Clinical and Communication Skills) • Our Community • Special Study Modules 1,2,3 (Family Case Project, Research Project, Community project)
Ethics	Here, two stations assessing student’s ethical decision making and ability to maintain patient confidentiality for specific case scenarios are assessed, aimed to gauge students’ sense of moral beliefs as well as a sense of their duty toward others. Competent students who take ethical concerns into consideration in these simulated scenarios may lead them to be better prepared to make decisions that are respectful, equitable and compassionate	• Personal and Professional Development • Patient Care (Clinical and Communication Skills)
Critical Thinking	In this station, student’s critical and analytical thought processes are assessed through a scenario involving a non-proven treatment declaration. issues and the current health care system. Students scoring well in this area would predict them to be competent in demonstrating critical awareness of current problems as well as analyse, synthesise and evaluate the literature. This would serve to be beneficial in developing their systematic critiques to form new hypotheses, expected in their research module	Special Study Module 2 (Research Project)

With this setting, the objectives of our study were to compare the MMI results of students who attended onsite and online MMI, compare the academic performance of the first-year result (first 2 semesters) of the students who passed the MMI, evaluate the effectiveness of the two MMI modes based on the academic performance of year one medical student. Furthermore, we also aimed to determine the effectiveness of onsite MMI in the evaluation of specific skills sets based on medical students’ performance across the first three years of medical school (as the students leave for partner medical schools for completion of the degree).

## Materials and methods

### Study setting and design

A retrospective study was conducted on medical students who successfully enrolled in the undergraduate medical programme at the PAPRSB Institute of Health Sciences (PAPRSB IHS) between 2016 and 2020 (Cohort 12 to 16). All methods were carried out in accordance with relevant guidelines and regulations. Being a retrospective study, there was no requirement for consent from any subjects and/or their legal guardian(s).

In the medicine programme admission process of the PAPRSB Institute of Health Sciences UBD, each MMI station tests a specific skill or knowledge in domains/themes, which includes the student’s ability to communicate and relay information such as reasons for wanting to do medicine, knowledge of current health system, environmental issues, and may also include scenarios about ethical issues, critical appraisal skills and other non-cognitive quality, evaluating student’s ability to communicate and personal or professional achievements. MMI allows the Institute to assess the attributes necessary to become a competent physician. In UBD, a combination of pre-university final results (from the A-level or International Baccalaureate) and MMI performance scores are used as the admission criteria for the medical programme, each carrying a 50% weightage to form an overall entrance score (International Advisory Board Document, 2017).

Student academic records were retrieved from the Examination Office records and their database from the Programme Leader was collected with permission. It is also mentioned in the next sentence: Participants’ age, gender, pre-university final results (A-level or equivalent), total and individual station MMI scores and examination results were collected from the Examination Office records. The examination results included students’ Grade Point Average (GPA), cumulative Grade Point Average (cGPA) from Health Sciences (HS), Our Community and Personal and Professional Development (OCPPD), Patient Care (PC) and Special Study Modules (SSM) 1, 2 and 3 throughout the three years of the student's undergraduate study.

On the day of the interviews, the Medicine programme leader met with all the interviewers to explain the station they would be evaluating. The interviewers were provided with a standardized station-specific rubric for scoring. Candidates were rated per given scenario at each station and scores were assigned based on performance. The interviewers had time to review and ask clarifying questions if required. The interviewers were recruited from the Institute of Health Sciences school's academic staff and adjunct lecturers.

A separate room was allotted for each station, and a single interviewer was assigned to each station. To ensure confidentiality the candidates were not informed about the stations in advance. On the day of the interview, all the candidates remained anonymous and were assigned unique candidate numbers for the interview. Interviewers had no access to the individual candidate’s admission packet before the interview.

The MMI was held as a face-to-face interview except for the year 2020, where it was conducted online due to the pandemic. The format and questions in the MMI themes were standardized in both on-site and online platforms. In the MMI, students were tested in ten individual stations that assessed the following skills:

- A: Students’ motivation, qualities and achievements, communication, and self-reflection
- B: Knowledge of Health Care and Current Issues
- C: Ethics
- D: Critical Thinking

Meanwhile, the examinations for each module (HS, PC and OCPPD) were held at the end of each semester, while SSM 1,2 and 3 assessments were held at the end of each year. Again, the pandemic in 2020 saw the examinations delivered online.

### Study population

All 140 students from the five cohorts of study years who successfully enrolled on the medicine programme (passed MMI and achieved required entrance scores) between 2016 and 2020 and completed the respective semesters of study during the course of the undergraduate medical programme were included in the study. This also had students who were terminated or withdrew after each academic year. Students with missing or incomplete data on any admission results were also excluded (Table 2). Cohort 12 – 15 had onsite MMI however cohort 16 students had a mixed format of online and onsite MMI.

**Table 2:** Number of students enrolled and withdrawn or terminated out of the programme during the period under study

Year	Number of Students Enrolled	Number of Students Withdrawn or Terminated
2016 (Cohort 12)	25	1
2017 (Cohort 13)	29	4
2018 (Cohort 14)	29	5
2019 (Cohort 15)	25	0
2020 (Cohort 16)	32	5

### Data collection

As only the grades of all modules were available, we first converted them into numeric marks by generating random variations within a normal distribution, using the already established mark ranges by the Medicine programme as the upper- and lower-mark limits. The same mark ranges have been consistently used for all student cohorts, namely: 85–100 (A +), 80–84.9 (A), 75–79.9 (B +), 70–74-9 (B), 65–69.9 (C +), 60–64.9 (C), and 0–59.9 (F). To reduce the variation for A + grade, a realistic upper limit mark of 91.9 was provided. Also, the exact marks for the three F grades found in the dataset were used. The average of each of the module block (HS, PC and OCCPD) were taken as a proxy for the student’s performance in each module block during the six semesters. Entrance scores were calculated using the formula: (0.5 × PUFR) + (0.5 × MMI score). As PUFR grade category differs by 20 points, all PUFR estimates were multiplied by 20 to facilitate result interpretation.

### Statistical analysis

For the dataset involving cohorts 12 to 15, the average of each of the module blocks (HS, PC and OCCPD) were taken as a proxy for the student’s performance in each module block during the six semesters. Kruskal–Wallis test was used to compare any differences in the median marks of each module between the 4 cohorts. Dunn’s post-hoc tests were conducted to identify any significant cohort-pairs for each significant result. Next, Spearman’s rank coefficient analysis was conducted to assess the direction of association and significance of MMI and each of the 4 stations A, B, C and D (the explanatory variables) on each respective module (HS, PC, OCPPD, SSM 1,2,3) and GPA scores (the outcome variables).

While for the dataset involving cohort 16, the average of each module block (HS, PC and OCCPD) were taken as a proxy for the student’s performance in each module block during the two semesters. Mann–Whitney’s test was used to compare any differences in the median marks of each module between the online and onsite groups. Scatterplots were drawn to determine any patterns between MMI marks and other variables for both groups. All statistical analyses were conducted using R statistical software (version 3.6.0). A *p*-value of < 0.05 was considered statistically significant.

### Ethical considerations

Ethical approval to conduct the study was provided by Institute of Health Science Research Ethics Committee and University Research Ethics Committee of UBD (ERC# UBD/PAPRSBIHSREC/2021/85).

## Results

### For cohorts 12 to 15

**Fig. 2:**
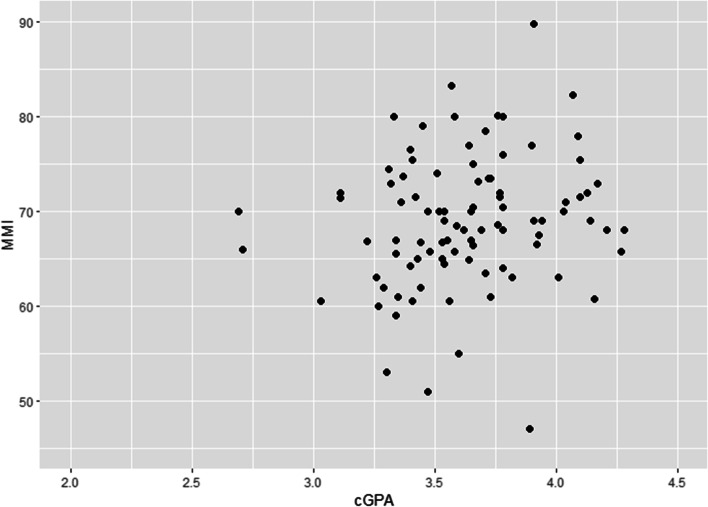
Scatterplot of cGPA and MMI marks from all students in cohorts 12 to 15

Our study population enrolled a total of 98 students from cohorts 12 to 15 of the undergraduate medical programme, but five were excluded from our study due to missing data. The overall median MMI score of all the students was 69.0 (IQR: 65.0 – 73.2) out of 100, with an overall median cGPA of 3.64 (IQR: 3.42 – 3.78) out of 5.0 (Table S1). A slightly positive relationship could be observed when examining the scatterplot of cGPA and MMI marks from the four cohorts (Fig. 2); this relationship became less apparent when examining the same scatterplot for each cohort (Figure S1).

Table 3 shows Spearman’s correlation test revealed positive and significant relationship between MMI and cGPA (rho = 0.23) and also for the GPA scores from the first 2 semesters (GPA1 rho = 0.25, GPA2 rho = 0.27). This observation is similar to that for station A (cGPA rho = 0.28, GPA1 rho = 0.34, GPA2 rho = 0.24). Interestingly, positive and significant relationship were also observed between MMI and GPA for other stations, but for the second year of medical school: station B (GPA4 rho = 0.25) and station D (GPA3 rho = 0.28, GPA4 rho = 0.24). With regards to specific modules, association results for MMI and station A tend to be similar in that positive and significant relationship could be observed for the first year of study (for HS1-2, PC1-2 & SSM1; Table 1 and Table S2). We found a positive and significant relationship between station B and SSM2 marks (rho = 0.23, *p* = 0.029). To some extent, we observed positive and significant relationship between station C and PC modules, and between station D and HS modules. Spearman’s correlation tests revealed positive and significant relationship between cGPA and overall MMI marks as well as for station A.

**Table 3:** Spearman’s correlation results between MMI and stations A-D marks (explanatory variables) with GPA and module marks (outcome variable), for cohorts 12 to 15

GPA & module marks as outcome variable:	Explanatory variables									
	MMI		Station A		Station B		Station C		Station D	
	rho	*p*-value	rho	*p*-value	rho	*p*-value	rho	*p*-value	rho	*p*-value
CGPA	0.23	**0.026**	0.28	**0.006**	0.15	0.138	0.09	0.389	0.16	0.123
GPA1	0.25	**0.017**	0.34	**< 0.001**	0.12	0.270	0.14	0.181	-0.06	0.58
GPA 2	0.27	**0.008**	0.24	**0.019**	0.09	0.399	0.20	0.053	0.02	0.885
GPA 3	0.13	0.209	0.08	0.432	0.10	0.346	-0.01	0.909	0.28	**0.007**
GPA 4	0.20	0.054	0.26	0.011	0.25	**0.015**	0.16	0.119	0.24	**0.021**
GPA 5	0.13	0.228	0.20	0.057	0.05	0.653	0.02	0.847	0.07	0.523
GPA 6	0.02	0.819	0.11	0.306	0.09	0.406	-0.07	0.533	0.12	0.256
Overall HS	0.12	0.235	0.18	0.087	0.16	0.131	0.06	0.577	0.06	0.567
Overall PC	0.22	**0.035**	0.23	**0.025**	0.12	0.257	0.21	**0.045**	0.12	0.254
Overall OC	0.18	0.078	0.28	**0.006**	0.10	0.340	0.10	0.318	-0.001	0.994
SSM1	0.21	**0.048**	0.37	** < 0.001**	0.17	0.111	0.14	0.176	0.14	0.166
SSM 2	0.11	0.280	0.20	0.052	0.23	**0.029**	0.09	0.390	0.20	0.058
SSM 3	0.02	0.845	0.02	0.844	0.11	0.316	-0.09	0.395	0.002	0.985

Bold values indicate statistical significance

### For cohort 16

**Fig. 3:**
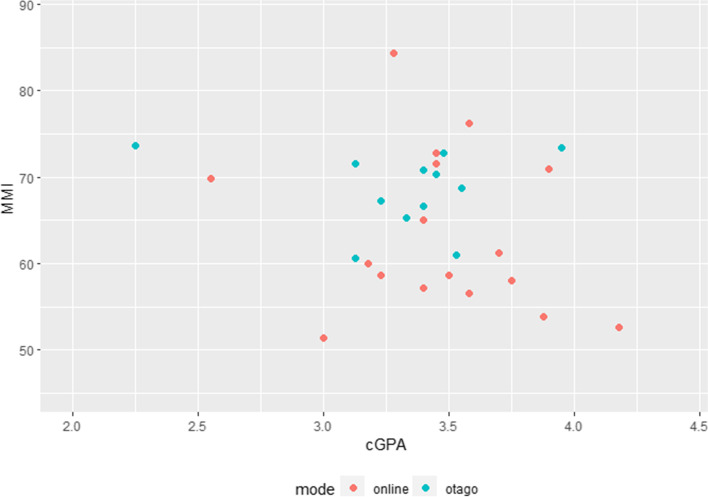
Scatterplot of cGPA and MMI marks for online and onsite groups (Cohort 16)

There was a total of 29 students enrolled to cohort 16, out of which 17 (58.6%) and 12 (41.4%) had undergone their MMI assessment through online and onsite modes, respectively. Their overall median MMI score was 66.6 (IQR: 58.6—71.6) out of 100, and their overall median cGPA was 3.45 (3.23—3.58) out of 5.0 (Table 4). No significant differences were observed when comparing the median marks between online and onsite MMI groups, except for station D where the online group scored significantly higher median marks than onsite MMI group (*p* = 0.040). No apparent relationships could be observed when examining the scatterplot of cGPA and MMI marks for both groups (Fig. 3).

**Table 4:** Student characteristics (cohort 16) for overall and by mode of MMI assessment (online and onsite)

Median marks (IQR)	Overall (*n* = 29)	online (*n* = 17)	Onsite (*n* = 12)	*p* value
mmi	66.6 (58.6—71.6)	60.0 (57.2—71.0)	69.5 (66.3—71.9)	0.066
station A	7.65 (7.04—8.02)	7.55 (7.00—8.20)	7.81 (7.13—8.02)	0.912
station B	6.50 (5.50—7.00)	6.50 (5.50—7.00)	6.50 (5.44—7.25)	0.532
station C	6.00 (6.00—7.00)	6.00 (6.00—7.00)	6.13 (6.00—6.50)	0.928
station D	6.00 (5.00—7.00)	7.00 (5.50—7.50)	5.25 (4.00—6.00)	**0.040**
GPA1	3.56 (3.33—3.83)	3.61 (3.44—3.83)	3.44 (3.11—3.71)	0.363
GPA2	3.32 (3.14—3.59)	3.36 (3.18—3.59)	3.28 (3.14—3.42)	0.549
CGPA	3.45 (3.23—3.58)	3.45 (3.28—3.70)	3.40 (3.21—3.49)	0.287
Overall HS	66.4 (62.5—69.3)	68.1 (64.5—69.8)	65.3 (60.1—67.9)	0.195
HS1	67.8 (64.0—69.1)	67.8 (66.0—68.8)	67.2 (61.4—70.0)	0.690
HS2	66.9 (61.2—69.5)	68.2 (61.5—71.9)	61.9 (59.6—67.1)	0.176
Overall PC	75.5 (72.9—76.6)	75.7 (74.1—77.4)	73.5 (70.8—75.7)	0.049
PC1	76.5 (74.1—78.8)	77.2 (75.9—80.0)	75.0 (71.8—76.9)	0.049
PC2	73.3 (70.7—75.8)	74.7 (72.2—76.2)	72.4 (69.5—75.1)	0.223
Overall OCPPD	73.3 (67.4—76.0)	73.5 (69.9—74.1)	69.5 (62.8—77.2)	0.413
OC1	77.4 (73.0—81.4)	76.8 (73.9—80.5)	78.7 (66.8—82.5)	0.929
OC2	67.7 (62.2—73.0)	68.4 (63.7—73.0)	64.5 (57.1—71.4)	0.352
SSM1	75.0 (70.0—81.0)	75.0 (69.0—82.0)	75.5 (71.8—78.3)	0.859

Bold values indicate statistical significance

## Discussion

Our findings indicate that the content design in MMI stations is essential and needs to be relevant to assess specific skills to ensure the selection of highly successful candidates in medical school. According to a review by Yusuf [2], MMI was positively correlated with cognitive, non-cognitive and performance-based assessments, including OSCE, argument ability, reasoning skills, simulation-based examination and communication skills, while a study done in Korea, [4] found a significant correlation in total MMI scores and academic achievement specifically for critical thinking and presentation skills. Our findings are also similar with a positive and significant relationship between MMI overall scores and the cGPA of students. Amongst the stations being tested, the station domain assessing students’ motivation, qualities and achievements, communication, and self-reflection were positively correlated with students’ cGPA. This observation was also seen for the first two semesters of their GPA and HS modules, as well as Special Study Module 1, also known as the family case study module. A study conducted by Eva et al., in 2012 also determined that students granted admission on the basis of MMI had better scores in the Canadian national licensing exam when compared with those who remained unsuccessful in the MMI [23].

This domain assessed the competencies of students’ responses and motivation when asked about the personal qualities of a good doctor as well as personal reasons for becoming one. This station also assessed students’ holistic high achievements in their previous school and other active involvement in community work, their ability to communicate as well as reflective thinking. Besides intelligence and a strong command of academia as shown by pre-university exam results, qualities such as ambition, dedication, passion, confidence, patience, motivation to learn, discipline, perseverance, good professional communication, teamwork, and altruism are assessed in the MMI. Such aspects are felt to be highly important in the selection process, which may ultimately lead to the success of an individual. While the ability to reflect can improve one’s self-regulation and broaden one perspective, it can also lead to improved performance, increased motivation and being ready to take on a new challenge [24]. Thus, it would come as no surprise that students scoring high in this domain would become high achievers, as observed in their cGPA results of this study in the subsequent years of the programme. Similar attributes have been observed by Cleland et al., who implemented a complete online MMI on medical undergraduate students [21].

It has been validated by a previous study conducted by Steinmayr et al., that intrinsic motivation and self-concept are key incremental factors for predicting academic success [25, 26].

It has been well-documented that GPA is the most consistent predictor of performance on multiple-choice question examinations in medical knowledge [9], as assessed in the HS module. A strong foundation in motivation, personal qualities and achievements, is also associated with higher scores in the Special Study Module 1 (SSM1) or family case study module, requiring students to undergo a longitudinal attachment to a family at various stages of the mother’s pregnancy, birth and post-natal care to learn about child development. Thus, having such qualities is not at all surprising to observe the success as demonstrated in this type of module. Besides cognitive abilities, a blend of personality characteristics may make it necessary to be successful in medical studies and eventually in the medical profession, as per the evidence that observed personality traits is one of the main non-intellectual variables predicting academic achievement in the higher education [27]. While students’ cognitive abilities and prior achievement are known to be among the best single predictors of academic success [28], studies have also supported that personality can predict academic performance where conscientiousness is consistently and positively related to first-year academic achievement, with specific qualities such as self-discipline and perseverance contributing to academic success [29]. Meanwhile, motivation can energise and direct one’s behaviour towards achievement and has been linked to being an important determinant of academic success [25]. Such skills, as assessed in the MMI, could thus predict higher academic performance, as seen in our students’ cGPA, as well as first-year modules as shown in the Health Sciences and SSM1.

It was interesting to observe that the station assessing students’ knowledge of the health care system was associated with better performance in SSM2, the research-based module. In this module, students are expected to perform research on a medical or health subject, where they would conduct data collection, analyse and interpret them.

With prior knowledge of the health care system, including its strengths and weaknesses, current health issues, and student experiences within the health services as assessed in this station, it is no surprise that students who scored well in this station would do so well in the SSM2 module. Also in this domain, awareness of current issues such as natural disasters and world events is assessed, as this would relate to having a better appreciation of community and population medicine including healthcare in the wider community, and also takes into account students’ prior shadowing or work experiences in health settings. Having awareness of current health issues not only indicates one’s maturity and appreciation of global issues but can potentially lead students to think strategically and proactively where this study has proven to be important as a predictor for good foundation in research skills. This has also been emphasized by Eva et al. [30].

For the station domain assessing ethics, which explores students’ ability to formulate ethical decisions on moral and legal issues, as well as managing a case regarding a confidentiality issue, we observed a positive and meaningful relationship with the Patient Care Module, which tests the student's clinical knowledge and skills using Objective Structured Clinical Examination (OSCE) stations. It has been well-researched that MMI is known to be the best predictor of OSCE performance [9] and for determining success in the medical school assessment [8]. This is because OSCE assesses areas that are most critical for performing skills such as application and conveying medical knowledge and procedural skills, with ethical decision-making skills playing a significant role in communication skills and breaking bad news. Practical professional ethics incorporate ethical principles into health care practices and medical decision-making. Ethical decision-making is one of the skills expected of medical students currently emphasised in clinical assessments [31]. This observation has led us to predict that students with competent ethical skills can be successful in clinical and communication areas, by taking ethical concerns into consideration, which can then lead them to be better prepared in making decisions that are respectful, equitable and compassionate, essential aspects of being a high-quality doctor. This is endorsed by the study conducted by Grone et al., which advocates that students having good clinical skills are better communicators as well [12].

The station assessing students’ ability to critically appraise a case study showed a correlation with students’ academic performance in the second year of study, the time they conducted the research-based module. In this module, students’ ability to analyse, synthesise and evaluate the literature, as well as equip themselves with research skills as expected in this research module sat during the second year, was observed from the correlation with the corresponding domain.

Thus, the domains assessed in our MMI stations serve to be important predictive factors to which areas (i.e., academic, clinical, research) they can succeed in, and are important components to be included in the selection criteria of medical students.

Studies have demonstrated that MMI could select candidates with high performance during medical training and was the most consistent predictor of success in early years at medical school across two separate cohorts [6], while Eva et al. (2012) [9] reported that highly performing candidates in MMI achieved high marks in a licensing national examination. Our study strongly suggests the importance of MMI in the selection process of successfully performing medical students as seen by our findings. High scores in students’ motivation, personal qualities and achievements predicted higher cGPA, health sciences in the first year as well as Special Study Module1. Good knowledge of health care as shown in their high performance in this particular MMI station predicted better grades in the research-based module. In contrast, strong ethical decision-making scores predicted better OSCE performance. This indicates that a strong MMI score during the student selection and entry process plays a role in the success of their academic performance in the 1^st^ year of medical school.

Our study is unique as it highlights the comparison of MMI results of students who attended onsite and online MMI along with the evaluation of overall GPA and the modules studied in the first year of Health Sciences (Medicine) by the two groups. Our results showed that students who appeared in face-2-face MMI achieved higher marks in MMI Station A but significantly low in D when compared with the online students. Interestingly, their CGPA remained almost the same. It is also observed that the onsite students did not obtain higher marks than the online MMI students in HS2, PC2 and OC2. Though the evidence is scarce concerning such comparison, Trustin Domes et al., as a part of their study, demonstrated the acceptability of virtual MMI with a G coefficient of 0.61, which was comparable with the synchronous MMIs [32]. This specifies that a strong MMI score during the student selection and entry process is required for efficacious academic performance and does not rely on the MMI mode. Further studies are warranted to assess the impact of online MMI on the early years of students’ academic achievement in graduate health professions. A comprehensive analysis comparing online MMI with traditional MMI formats, considering the entire pool of qualified candidates, would determine the superiority.

### Study limitations and future research

This study had several limitations. The small number of students enrolled each year is the foremost limitation, which makes it difficult to perform multivariant analysis. However, this will help us continue collect data for future research. Each MMI station's degree of difficulty and reliability could also have contributed to biases. Internet lagging or disruption could have interfered with students' MMI performance. Our study involves a single institution, and it is unknown to what extent these findings can be generalised beyond the PAPRSB Institute of Health Sciences. Besides students leaving for partner medical schools and moving to a new earning environment limits us from correlating MMI results with their final year results.

It will be interesting to observe whether our MMI can predict future performance in examinations beyond the third year of medical school. It will also be interesting to watch whether it can predict student dropout rates, future professional behaviour and resilience, all of the qualities that the MMI claims to measure at medical school intake.

## Conclusion

Our study is unique as it highlights a comparison of MMI results of students who attended onsite and online MMI, along with the evaluation of overall GPA and the modules studied in the first year of Health Sciences (Medicine). Furthermore, our study validates the acceptability of virtual MMI. As a preliminary study our study indicates, a good MMI score achieved during the student selection and entry process is a predictor of efficacious academic performance and is not reliant on the mode of MMI however, more research on a larger cohort is essential to support this evidence. Further studies are warranted to assess the impact of online MMI on the early years of students’ academic achievement in graduate health professions. A comprehensive analysis comparing online MMI with traditional MMI formats, considering the entire pool of qualified candidates, would determine the superiority.

### Recommendation

The outcomes from this study enabled the BHSc Medicine programme to review the selection criteria design of MMI and programme curriculum to keep up with current medical educational trends. Given some of the student failures seen in the programme, this study's results may identify areas that may make the interview selection stringent to enhance the matriculated candidates' success.

## Supplementary Information

**Additional file 1.**

## Data Availability

All data generated or analysed during this study are included in this article [and its supplementary information files].
