# Supplementary material for: Multiple mini-interviews is a predictor of students’ academic achievements in early undergraduate medical years: a retrospective study
Source: BMC Med Educ. 2023 Mar 27;23:187. doi: 10.1186/s12909-023-04183-7 (PMC10044430; doi:10.1186/s12909-023-04183-7)
Supplement: Supplementary file 1 — Additional file 1. [file 12909_2023_4183_MOESM1_ESM.docx]

***APPENDIX:***

***SUPPLEMENTARY DATA:***

***Table S1.*** Students’ (cohorts 12 to 15) characteristics: Overall and Stratified by Each Cohort

| Median marks (IQR) | Overall (n=93) | Cohort 12 (n=23) | Cohort 13 (n=23) | Cohort 14 (n=24) | Cohort 15 (n=23) | | p-value |
| --- | --- | --- | --- | --- | --- | --- | --- |
| MMI | 69.0 (65.0 - 73.2) | 69.0 (62.0 - 72.5) | 69.0 (66.0 - 73.3) | 66.8 (64.7 - 74.0) | 70.0 (66.9 - 72.4) | | 0.798 |
| station A | 7.3 (6.7 - 7.7) | 7.1 (6.5 - 7.5) | 7.2 (6.8 - 7.4) | 7.5 (6.7 - 7.9) | 7.5 (7.1 - 7.9) | | 0.118 |
| station B | 6.5 (5.8 - 7.2) | 6.5 (5.8 - 7.0) | 6.5 (5.9 - 7.0) | 6.8 (5.8 - 7.8) | 6.5 (6.0 - 7.0) | | 0.545 |
| station C | 6.5 (6.0 - 7.3) | 6.3 (5.3 - 6.8) | 6.5 (6.1 - 7.5) | 6.3 (5.5 - 7.0) | 7.0 (6.6 - 7.6) | | **0.003ʱʸ** |
| station D | 6.5 (5.0 - 8.0) | 7.5 (6.5 - 8.5) | 7.5 (6.5 - 8.5) | 6.5 (4.5 - 8.0) | 5.0 (4.5 - 5.8) | | **<0.001ʱʷ** |
| cGPA | 3.64 (3.42 - 3.78) | 3.77 (3.41 - 3.92) | 3.54 (3.43 - 3.67) | 3.55 (3.39 - 3.76) | 3.68 (3.52 - 3.96) | | 0.138 |
| GPA1 | 3.61 (3.39 - 3.94) | 3.67 (3.42 - 4.05) | 3.44 (3.28 - 3.59) | 3.47 (3.21 - 3.94) | 3.94 (3.72 - 4.20) | | **<0.001ʷʸ** |
| GPA2 | 3.50 (3.23 - 3.83) | 3.59 (3.36 - 3.87) | 3.41 (3.25 - 3.66) | 3.14 (2.83 - 3.52) | 3.82 (3.57 - 4.16) | | **<0.001^ʷʸ** |
| GPA3 | 3.89 (3.56 - 4.17) | 4.11 (3.53 - 4.42) | 3.78 (3.61 - 4.17) | 3.84 (3.56 - 4.00) | 3.78 (3.50 - 3.94) | | 0.239 |
| GPA4 | 3.54 (3.29 - 3.79) | 3.58 (3.38 - 3.86) | 3.50 (3.27 - 3.65) | 3.63 (3.38 - 3.92) | 3.46 (3.27 - 3.71) | | 0.427 |
| GPA5 | 4.00 (3.69 - 4.13) | 4.00 (3.75 - 4.13) | 3.81 (3.53 - 4.13) | 3.88 (3.70 - 4.03) | 4.06 (3.91 - 4.22) | | 0.053 |
| GPA6 | 3.33 (3.06 - 3.72) | 3.50 (2.92 - 3.83) | 3.39 (3.14 - 3.53) | 3.28 (3.10 - 3.59) | 3.28 (3.03 - 3.78) | | 0.995 |
| Overall HS | 69.5 (67.1 - 74.2) | 72.2 (65.9 - 77.5) | 69.1 (66.8 - 71.0) | 69.1 (66.2 - 71.5) | 71.8 (68.1 - 75.0) | | 0.264 |
| HS1 | 73.0 (69.0 - 79.7) | 71.0 (67.5 - 80.0) | 70.0 (67.5 - 72.5) | 72.8 (66.9 - 77.8) | 80.7 (75.0 - 84.5) | | **<0.001ʱʷʸ** |
| HS2 | 69.0 (64.0 - 74.0) | 69.0 (63.0 - 75.0) | 67.0 (63.0 - 70.5) | 67.3 (64.4 - 72.1) | 72.5 (69.9 - 79.4) | | **0.021ʷ** |
| HS3 | 73.8 (69.4 - 79.8) | 79.4 (64.7 - 84.5) | 75.4 (72.5 - 80.0) | 73.7 (68.9 - 79.5) | 71.7 (69.5 - 75.2) | | 0.154 |
| HS4 | 68.0 (64.0 - 72.3) | 71.0 (64.0 - 76.0) | 70.2 (67.1 - 73.2) | 67.0 (62.9 - 69.2) | 65.3 (60.8 - 69.4) | | **0.015ʷ** |
| HS5 | 70.8 (67.1 - 74.0) | 70.1 (64.7 - 74.0) | 70.4 (67.3 - 75.3) | 68.0 (65.6 - 71.1) | 72.7 (71.0 - 75.8) | | **0.003ʸ** |
| HS6 | 65.8 (61.2 - 74.1) | 74.8 (65.7 - 80.6) | 61.2 (57.2 - 64.6) | 66.1 (62.2 - 70.3) | 67.1 (63.6 - 73.3) | | **<0.001*ʷ** |
| Overall PC | 74.3 (72.1 - 77.4) | 72.8 (71.5 - 75.3) | 75.1 (73.2 - 76.4) | 73.1 (69.5 - 77.2) | 77.1 (74.1 - 79.1) | | **0.006ʱʸ** |
| PC1 | 75.1 (71.4 - 79.6) | 75.0 (71.0 - 79.0) | 75.0 (72.5 - 77.0) | 71.7 (68.3 - 79.0) | 78.6 (76.2 - 81.0) | | **0.009ʸ** |
| PC2 | 73.1 (68.0 - 78.0) | 68.0 (65.0 - 71.0) | 76.0 (73.5 - 78.5) | 69.0 (65.3 - 74.4) | 78.3 (74.1 - 82.2) | | **<0.001*ʱˠʸ** |
| PC3 | 76.2 (71.5 - 81.3) | 81.5 (79.8 - 82.7) | 72.3 (69.4 - 77.0) | 74.1 (70.9 - 80.0) | 74.2 (71.6 - 78.3) | | **<0.001*^ʱ** |
| PC4 | 76.5 (73.0 - 80.0) | 76.0 (74.0 - 79.0) | 75.5 (71.9 - 79.4) | 76.5 (69.2 - 81.8) | 77.6 (75.8 - 79.9) | | 0.71 |
| PC5 | 75.1 (71.0 - 78.5) | 74.0 (69.0 - 77.0) | 76.5 (73.3 - 78.4) | 72.9 (67.7 - 77.1) | 77.7 (73.9 - 81.2) | | **0.007ʱʸ** |
| PC6 | 73.9 (69.4 - 76.6) | 67.9 (63.9 - 71.0) | 75.4 (73.7 - 81.4) | 74.9 (70.9 - 78.5) | 75.1 (71.4 - 76.5) | | **<0.001*^ʱ** |
| Overall OCPPD | 76.3 (72.5 - 78.5) | 76.3 (74.4 - 79.0) | 75.6 (72.9 - 77.1) | 76.1 (71.1 - 78.4) | 77.4 (75.1 - 80.4) | | 0.127 |
| OC1 | 72.0 (68.0 - 76.0) | 75.0 (72.0 - 78.0) | 71.0 (67.0 - 72.0) | 70.3 (65.9 - 74.3) | 73.8 (72.3 - 79.3) | | **<0.001*^ʷʸ** |
| OC2 | 73.2 (68.4 - 79.0) | 68.0 (65.5 - 76.0) | 76.0 (72.0 - 80.5) | 73.8 (68.7 - 78.4) | 73.2 (69.9 - 75.3) | | **0.013*** |
| OC3 | 78.4 (72.3 - 83.4) | 74.9 (70.8 - 78.2) | 79.7 (72.7 - 86.1) | 78.9 (76.1 - 85.8) | 81.0 (75.6 - 83.9) | | 0.052 |
| OC4 | 75.7 (69.4 - 83.0) | 84.0 (77.5 - 86.0) | 70.4 (69.4 - 73.8) | 80.9 (64.8 - 84.0) | 73.0 (69.1 - 76.0) | | **<0.001*ʱ** |
| OC5 | 78.5 (75.7 - 82.0) | 81.0 (77.0 - 82.0) | 75.2 (70.8 - 76.4) | 77.0 (75.3 - 80.8) | 83.6 (80.3 - 86.5) | | **<0.001*ʷʸ** |
| OC6 | 77.5 (73.9 - 83.1) | 77.3 (73.4 - 82.2) | 76.4 (74.4 - 83.6) | 74.5 (70.3 - 78.6) | 83.5 (77.0 - 86.2) | | **0.001ʸ** |
| SSM1 | 74.9 (71.5 - 79.3) | 77.4 (74.5 - 78.5) | 72.9 (70.2 - 77.1) | 72.6 (68.0 - 80.3) | 74.9 (72.4 - 80.8) | | 0.115 |
| SSM2 | 70.9 (68.5 - 74.1) | 71.9 (69.6 - 75.3) | 70.6 (68.7 - 72.9) | 71.1 (68.6 - 74.4) | 70.2 (66.7 - 74.3) | | 0.351 |
| SSM3 | 83.0 (80.0 - 88.6) | 84.0 (80.0 - 86.5) | 78.9 (78.6 - 87.1) | 87.9 (86.6 - 89.6) | 81.4 (80.4 - 83.0) | | **<0.001^ˠʸ** |
| Significant differences for cohort 12-13*, 12-14^, 12-15ʱ, 13-14ˠ, 13-15ʷ, and 14-15ʸ pairs | | | | | |  |  |

***Table S2.*** *Spearman’s correlation results between MMI and stations A-D marks (explanatory variables) with module marks (outcome variable), for cohorts 12 to 15.*

| Module marks as outcome variable: | Explanatory variables | | | | | | | | | |
| --- | --- | --- | --- | --- | --- | --- | --- | --- | --- | --- |
|  | MMI | | Station A | | Station B | | Station C | | Station D | |
|  | rho | p-value | rho | p-value | rho | p-value | rho | p-value | rho | p-value |
| Overall HS | 0.12 | 0.235 | 0.18 | 0.087 | 0.16 | 0.131 | 0.06 | 0.577 | 0.06 | 0.567 |
| HS1 | 0.21 | **0.047** | 0.31 | **0.002** | 0.16 | 0.118 | 0.18 | 0.083 | -0.10 | 0.331 |
| HS2 | 0.25 | **0.016** | 0.21 | **0.043** | 0.07 | 0.504 | 0.15 | 0.157 | -0.15 | 0.149 |
| HS3 | 0.08 | 0.439 | 0.01 | 0.928 | 0.06 | 0.593 | -0.02 | 0.860 | 0.25 | **0.015** |
| HS4 | 0.20 | 0.058 | 0.16 | 0.115 | 0.25 | **0.016** | 0.13 | 0.206 | 0.27 | **0.010** |
| HS5 | 0.06 | 0.588 | 0.15 | 0.162 | 0.08 | 0.475 | 0.05 | 0.625 | 0.03 | 0.769 |
| HS6 | -0.08 | 0.433 | 0.02 | 0.818 | 0.06 | 0.553 | -0.21 | **0.039** | -0.04 | 0.714 |
| Overall PC | 0.22 | **0.035** | 0.23 | **0.025** | 0.12 | 0.257 | 0.21 | **0.045** | 0.12 | 0.254 |
| PC1 | 0.16 | 0.119 | 0.23 | **0.029** | -0.01 | 0.956 | 0.15 | 0.139 | -0.01 | 0.929 |
| PC2 | 0.29 | **0.021** | 0.22 | **0.033** | 0.09 | 0.400 | 0.26 | **0.011** | -0.01 | 0.935 |
| PC3 | 0.10 | 0.333 | -0.01 | 0.921 | 0.12 | 0.241 | -0.06 | 0.561 | 0.30 | **0.004** |
| PC4 | 0.02 | 0.827 | 0.14 | 0.191 | 0.01 | 0.908 | 0.08 | 0.425 | 0.20 | 0.061 |
| PC5 | 0.15 | 0.154 | 0.18 | 0.081 | 0.06 | 0.558 | 0.16 | 0.124 | -0.02 | 0.819 |
| PC6 | 0.19 | 0.075 | 0.21 | **0.045** | 0.12 | 0.270 | 0.27 | **0.010** | 0.07 | 0.511 |
| Overall OC | 0.18 | 0.078 | 0.28 | **0.006** | 0.10 | 0.340 | 0.10 | 0.318 | -0.001 | 0.994 |
| OC1 | 0.14 | 0.177 | 0.18 | 0.091 | 0.18 | 0.086 | 0.07 | 0.491 | -0.04 | 0.702 |
| OC2 | 0.19 | 0.072 | 0.15 | 0.157 | -0.03 | 0.808 | 0.10 | 0.326 | -0.07 | 0.514 |
| OC3 | 0.12 | 0.266 | 0.17 | 0.097 | 0.12 | 0.233 | 0.04 | 0.725 | -0.01 | 0.900 |
| OC4 | 0.01 | 0.933 | 0.12 | 0.264 | 0.07 | 0.493 | -0.02 | 0.815 | 0.18 | 0.087 |
| OC5 | 0.12 | 0.248 | 0.20 | 0.053 | 0.06 | 0.567 | 0.04 | 0.680 | -0.16 | 0.130 |
| OC6 | 0.08 | 0.432 | 0.17 | 0.107 | -0.02 | 0.824 | 0.13 | 0.218 | 0.03 | 0.797 |
| Bold values indicate statistical significance | | | | | | | | | |  |

***
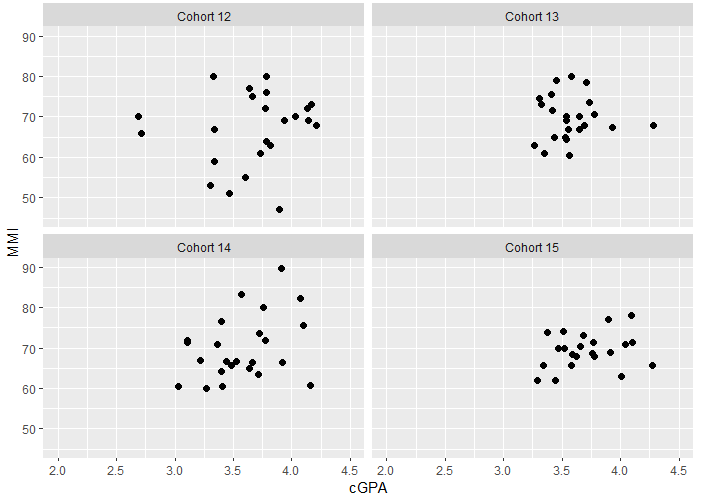
***

***Figure S1. Scatterplot of cGPA and MMI marks from students in each cohort (Cohorts 12 to 15)***
